# Supplementary material for: The interactome of intact mitochondria by cross-linking mass spectrometry provides evidence for coexisting respiratory supercomplexes
Source: Mol Cell Proteomics. 2017 Dec 8;17(2):216–32. doi: 10.1074/mcp.RA117.000470 (PMC5795388; doi:10.1074/mcp.RA117.000470)
Supplement: Supplemental Data [file supp_RA117.000470_133922_0_supp_23978_fzffwf.zip › spectra_annotation/mito_spectra_annotation/472-1-5-1-11-1.pdf]

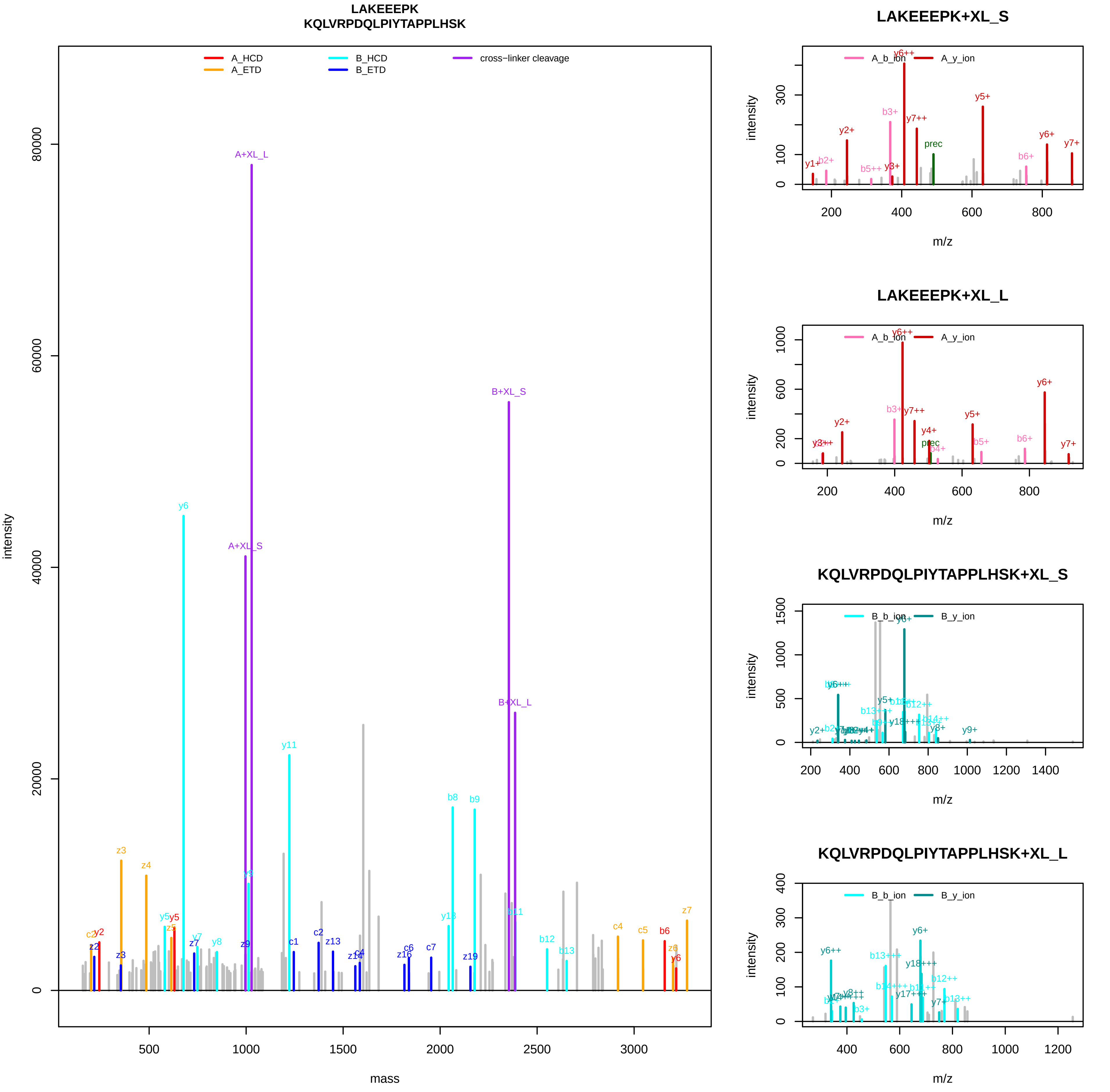

LAKEEEPK
KQLVRPDQLPIYTAPPLHSK
A_HCD
A_ETD
B_HCD
B_ETD
cross−linker cleavage
A+XL_L
A+XL_S
B+XL_S
B+XL_L
y6
y11
y9
b8
b9
b11
b12
b13
y13
y5
y7
y8
z3
z4
z5
z7
z9
z13
z14
z16
z19
c1
c2
c4
c5
c6
c7
b6
z6
z7
intensity
mass
LAKEEEPK+XL_S
LAKEEEPK+XL_L
KQLVRPDQLPIYTAPPLHSK+XL_S
KQLVRPDQLPIYTAPPLHSK+XL_L
A_b_ion
A_y_ion
B_b_ion
B_y_ion
prec
m/z
